# Hypercapnia in hospitalized children and adolescents with anorexia nervosa as a predictive marker for readmission: a prospective study

**DOI:** 10.1007/s40519-023-01624-6

**Published:** 2023-11-03

**Authors:** Pedro Viaño-Nogueira, Cristina Aparicio-López, Ángela Prieto-Campo, Goretti Morón-Nozaleda, Ricardo Camarneiro-Silva, Montserrat Graell-Berna, Carmen de Lucas-Collantes

**Affiliations:** 1 Department of Pediatrics, Universidad Autónoma de Madrid, Madrid, Spain; 2 Department of Nephrology, Hospital Infantil Universitario Niño Jesús, Madrid, Spain; 3 Statistics and Methodology Unit, Galicia Sur Health Research Institute (ISS Galicia Sur), SERGAS-UVIGO, Vigo, Spain; 4 Department of Psychiatry, Hospital Infantil Universitario Niño Jesús, Madrid, Spain

**Keywords:** Anorexia nervosa, Feeding and eating disorders, Hospital readmission, Hypercapnia, Medical complications, Respiratory acidosis

## Abstract

**Purpose:**

To determine whether hypercapnia is associated with risk of hospital readmission related to anorexia nervosa (AN) in children and adolescents.

**Methods:**

We performed a prospective study of patients ≤ 18 years old admitted due to AN decompensation from November 2018 to October 2019. Both subtypes of AN, restricting subtype (AN-R) and binge-eating/purging subtype (AN-BP), were included. Study participants were evaluated upon admission, at discharge and six months after discharge. *T*-tests or Mann–Whitney *U* tests was used to compare means values. Pearson or Spearman correlations were used to measure the association between two variables. Logistic regression models were developed to evaluate the relationship between scoring methods and readmission.

**Results:**

Of the 154 persons admitted during the study period, 131 met the inclusion criteria. Median age was 15.1 years. At admission, 71% of participants were malnourished and 33 (25%) had been previously admitted.

We observed a marked decrease in venous pH and stable pCO_2_ elevation during follow-up period. Hypercapnia at discharge was associated with a twofold increased likelihood of readmission and the odds of readmission increased as discharge pCO_2_ rose. These findings did not depend on AN subtype or participant sex. Electrolytes persisted within the normal range.

**Conclusion:**

Hypercapnia and respiratory acidosis are common alterations in children and adolescents hospitalized due to AN decompensation. Hypercapnia persists for at least 6 months after discharge despite clinical improvement and is associated with higher odds of readmission. This is the first study to identify an abnormal laboratory finding as a potential predictor of readmission in AN.

**Level of evidence:**

IV: Multiple time series without intervention.

## Introduction

Anorexia nervosa (AN) is a severe eating disorder (ED) due to multifactorial causes and with considerable psychiatric and physiologic comorbidities [1]. It is characterized by a marked restriction of food consumption causing weight loss, an intense fear of gaining weight and a self-image dysperception [2]. Cases of AN may be classified as restricting (AN-R) and binge-eating and purging (AN-BP) subtypes, based on the occurrence of binge-eating episodes or purging behavior over the previous 3 months [2].

AN often arises in adolescence [3]. Its incidence among teenagers younger than 15 years of age has increased in recent decades, although it is unclear whether this rise is due to earlier diagnosis or earlier disorder onset [4]. More than 90% of adults with disease are female [5], although this proportion may be lower in children and adolescents [6]. Mental disorders coexist in more than 70% of cases [7, 8], especially anxiety and mood and personality disorders [3, 5, 8, 9]. As many as one in three patients with AN may present autism spectrum disorder [10–12].

AN-associated mortality ranges from 2 to 8%, the highest mortality rate for all psychiatric disorders [7, 13], mainly resulting from suicide and medical complications [1, 14, 15]. Mortality during the initial stages of medical stabilization is proportional to the degree of malnutrition [16] and is correlated with the use of intensive care facilities [17].

AN affects every system of the body due to complications resulting from fasting and malnutrition [18]. Organic alterations may differ with individual eating and purging behaviors [19]. Hypokalemia, a potentially lethal electrolyte disturbance, is almost exclusively associated with active purging behavior [20]. Metabolic alkalosis has been classically linked to a depletion of hydrogen caused by vomiting and use of diuretics [21]. Hypercapnia (pCO_2_ over 45 mmHg) has been described in adolescents hospitalized due to AN and improves from admission to discharge [22]. An increased vagal tone and/or decreased respiratory muscle power could be the causes of hypercapnia. Sarcopenia is a frequent finding that leads to weakness, impaired diaphragm function and pulmonary malfunction [23, 24].

Treatment in the outpatient setting, as opposed to hospitalization, is preferred for persons diagnosed with AN. Multidisciplinary teams, comprising physicians and specialists in psychological, psychiatric, and nutritional care, are considered crucial to recovery [25–28]. Family-based treatment is a common approach for children and adolescents with AN [29], although the evidence supporting this care modality has not been challenged [30]. Hospitalization is frequently required when acute medical stabilization is needed or treatment adherence is not guaranteed [25, 31, 32].

Readmissions in AN are common. Nevertheless, readmissions as an outcome itself have been scarcely studied [33–35]. Repeat hospitalizations have been reported to seriously disrupt child and adolescent development and familial functioning [36]. Previous hospitalizations, low socioeconomic status, poor weight gain during earlier admissions and younger age at diagnosis have been found to be indicators of readmission risk [37, 38]. Thus far, however, no physiologic abnormalities have been linked to the probability of readmission.

The aim of this study was to determine the abnormal clinical and laboratory alterations findings associated with risk of AN-related hospital readmissions in children and adolescents.

## Methods

### Study design and setting

A prospective study was performed from November 2018 to October 2019 in the Eating Disorder Ward of “Hospital Infantil Universitario Niño Jesús”, a publicly funded children's hospital in Madrid, Spain. The study protocol was approved by both the hospital research ethics committee and hospital administrator in September 2018 (registration number R-0046/18). Data were anonymized to prevent patient identification. The study was conducted in accordance with the Declaration of Helsinki.

Participants were assessed at three time points: upon admission (*t*_0_), at discharge (*t*_1_) and 6 months after discharge (*t*_2_). No post-discharge visit was scheduled in 14 patients who underwent clinical follow-up in other facilities.

### Participants

The study group consisted of every child and adolescent ≤ 18 years of age admitted to the eating disorders ward who met at that time the diagnostic criteria for AN from the Diagnostic and Statistical Manual of Mental Disorders, fifth edition (DSM-5) [2]. Both subtypes (AN-R and AN-BP) were included.

Participants were referred for hospitalization from the day hospital, emergency department or other hospitals and specialist clinics. The decision to hospitalize was mainly based on the physiologic impact secondary to malnutrition and purging, according to the recommendations of the American Psychiatric Association and the Society for Adolescent Medicine [32, 39, 40].

Inclusion criteria for the study were as follows: (1) hospitalization due to AN and (2) signed informed consent provided by parents and, in the case of patients over 12 years of age, the patients themselves. Children and adolescents with an acute or chronic lung disease or chronic kidney disease were excluded.

Every participant served as their own control throughout the study.

### Variables

Clinical information gathered for the study was divided into three categories:

1. Demographic and anthropometric data: age, sex, length of hospital stay, weight, height and body mass index (BMI). These data were collected upon admission, at discharge and 6 months after discharge. Severity of malnutrition was expressed as the number of standard deviations below the mean BMI is (*Z*-score) for each participant’s age and sex, according to the 2010 Comprehensive Spanish Growth Studies [41, 42].
2. Information related to AN: maximum weight, rate of weight loss (if any), duration of the ED before admission, number of previous admissions related to eating disorders and presence and characteristics of purging behaviors.
3. Clinical and laboratory findings: heart rate, systolic and diastolic blood pressure and electrocardiogram (ECG), in addition to a complete physical examination. Blood samples were obtained in the morning after an 8-h fasting period, both upon admission and at discharge. Analyses included complete blood count, electrolytes, glucose, test of kidney and liver tests and venous blood gases.

### Statistical analysis

Continuous variables were expressed using mean values (M) and standard deviations (SD) or median, interquartile range (IQR) and range, based on normality. Variables were assessed for normality using the Kolmogorov–Smirnov tests.

The Student’s *T*-test for independent samples or Mann–Whitney *U* test was used to compare mean values between two groups. *T*-tests for paired samples were used when comparing dependent groups. For three or more groups, we used one-factor analysis of variance (ANOVA) or the Kruskal–Wallis test.

Pearson correlations or Spearman correlations were used for measuring the strength and direction of association between two variables. For three or more variables, multiple linear regressions were used.

Logistic regression models were developed to analyze the relationship between the scoring methods in question (i.e., BMI *Z*-score, venous pH, partial pressure of carbon dioxide (pCO_2_) bicarbonate concentration (HCO_3_), on admission and at discharge) and readmission.

*P*-values less than 0.05 were considered statistically significant. Statistical analyses were performed using IBM SPSS Statistics (version 28).

## Results

One hundred fifty-four individuals were admitted during the study period, of whom 131 met the inclusion criteria. Twenty patients were admitted for an ED other than AN. Three patients declined to participate. No patients were excluded because of a previous lung disease.

The demographic and clinical variables of the study sample are shown in Table 1. At admission, 71% of participants were malnourished (BMI *Z*-score ≤ − 1 SD for sex and age). Six participants (4%) reported that they drank over 2 L of water per day (range 3–6 L). Only one participant (0.7%) acknowledged using drugs (laxatives) to lose weight.

**Table 1:** Demographic and medical characteristics of the sample (*N* = 131)

Male/female (*N*)	8/123
AN-R/AN-BP (*N*)	22/109
Length of hospitalization (days)	29 (26–36)
On admission:	
Age (years)	15.1 (13.5–16.4)
Time elapsed from initial thoughts/behaviors related to AN (months)	11.8 (7.4–27.4)
Previous admissions related to ED:	
None	98 (74.8%)
One	21 (16%)
Two	5 (3.8%)
Three	5 (3.8%)
Four	1 (0.8%)
Five	1 (0.8%)

Table values are median (interquartile range) for continuous variables, ratio for dichotomous variables and n (column %) for categorical variables

*AN* anorexia nervosa, *AN-BP* anorexia nervosa—binge-eating/purging subtype, *AN-R* anorexia nervosa—restricting subtype, *ED* eating disorder

Thirty-three participants (25%) had previous admissions due to an ED, including those treated in other facilities. Five had been admitted twice and 19 participants (12%) were readmitted during the study period due to clinical deterioration. Eleven participants (58%) had never been admitted before the study period.

Weight and BMI significantly increased from admission to discharge (Table 2).

**Table 2:** Anthropometric and clinical information of the sample (*N* = 131)

	Admission (*t*_0_)	Discharge (*t*_1_)	6 months post-discharge (*t*_2_)	*p*-value *t*_0_ − *t*_1_	*p*-value *t*_0_ − *t*_2_	*p*-value *t*_1_ − *t*_2_
Weight (kg)	41.8 (36.4 to 46.8)	46.8 (43.2 to 51.2)	48.8 (45.7 to 53.1)	** < 0.0001**	** < 0.0001**	** < 0.0001**
Percentage of weight loss before admission	21.2 (27 to 16)	–	–	–	–	–
BMI (kg/m^2^)	16.2 (14.7 to 17.5)	18.2 (17.2 to 19.1)	18.7 (17.7 to 20.1)	** < 0.0001**	** < 0.0001**	** < 0.0001**
Weight *Z*-score	− 1.1 (− 1.7 to − 0.7)	− 0.7 (− 1.1 to − 0.3)	− 0.5 (− 1.1 to − 0.2)	** < 0.0001**	** < 0.0001**	** < 0.0001**
BMI *Z*-score	− 1.4 (− 1.8 to − 0.9)	− 0.8 (− 1.1 to − 0.6)	− 0.7 (− 1.1 to − 0.3)	** < 0.0001**	** < 0.0001**	** < 0.0001**
HR (beats/minute)	58 (49 to 69)	77 (67 to 82)	75 (61 to 86)	** < 0.0001**	** < 0.0001**	0.537
SBP (mmHg)	98 (91.8 to 108)	100 (95 to 105)	105.5 (98 to 112)	0.54	**0.004**	**0.001**
DBP (mmHg)	60 (55 to 66)	58 (54 to 62)	61.5 (59 to 71)	** < 0.0001**	0.121	**0.004**

Table values indicate medians (interquartile range) for continuous variables

*BMI* body mass index, *DBP* diastolic blood pressure, *HR* heart rate, *SBP* systolic blood pressure

Bold indicates *p* < 0.05

Heart rate (HR) increased significantly during hospitalization. 47% of participants presented bradycardia (HR < 60 bpm) upon admission, as compared to 8% at discharge (Table 2). No participants were diagnosed with arrhythmia other than sinus bradycardia based on the findings of clinical and electrocardiographic assessment. HR was slightly but significantly correlated with pCO_2_, both upon admission (*r* = − 0.27, *p* = *0.0*01) and at discharge (*r* = − 0.23, *p* = 0.023). No patient received supplementary oxygen therapy or intravenous fluids during their hospital stay.

While 14% of participants showed hypotension (systolic blood pressure -SBP- < 90 mmHg) at the beginning of hospitalization and 8% at the end of hospitalization, systolic and diastolic blood pressure did not significantly differ from admission to discharge.

Venous pH, pCO_2_ and HC${\text{O}}_{3}^{ - }$ values remained stable throughout the follow-up period, despite the remarkable weight increase and the clinical progress (Table 3). However, hypercapnia at discharge was associated with a 2.4-fold increased likelihood of readmission.

**Table 3:** Venous blood gas results of the sample (N = 131)

	Admission (*t*_0_)	Discharge (*t*_1_)	6 months from discharge (*t*_2_)	*p*-value *t*_0_–*t*_1_	*p*-value *t*_0_–*t*_2_	*p*-value *t*_0_–*t*_2_	Reference range
pH	7.34 (7.32–7.37)	7.33 (7.31–7.36)	7.33 (7.32–7.37)	**0.037**	0.304	**0.033**	7.35–7.45
pCO_2_	52 (48–56)	55 (50–59)	55 (50–57)	**0.02**	0.796	0.147	35–45
HC${\text{O}}_{3}^{ - }$	28.3 (26.5–29.7)	29 (27.1–30.2)	28.6 (26.4–29.9)	0.11	0.834	0.837	21–29
pCO_2_ > 45 mmHg and pH < 7.35 (%)	67 (51.2)	84 (64.1)	79 (60.3)	–	–	–	–
pCO_2_ > 45 mmHg (%)	111 (84.7)	112 (85.5)	110 (84.0)	–	–	–	–

Table values indicate medians (interquartile range) for continuous variables and n (column %) for categorical variables

HC${\text{O}}_{3}^{ - }$, bicarbonate concentration; pCO_2_, partial pressure of carbon dioxide

Bold indicates *p* < 0.05

A logistic regression model was designed to measure the effect of pCO_2_ at discharge and on readmission. BMI Z-score for sex and age at discharge and pCO_2_ at discharge were included as independent variables. Readmission was included as the dependent variable. The logistic regression model significantly (Table 4) showed a significant, 1.16-fold increase in the odds of readmission with every 1-mmHg increase in pCO_2_ at the time of discharge (*p* = 0.03). The readmission rate of patients who presented hypercapnia upon discharge was higher than that of patients with no hypercapnia, albeit not significantly (18/94 − 16.1%- versus 1/18 − 5.3%-, *p* = 0.31). However, a higher BMI *Z*-score for sex and age was not significantly associated with lower odds of readmission.

**Table 4:** Association between predictors and hospital readmission assessed by logistic regression

	*b*	S.E	*p*	OR for readmission
pCO_2_ upon admission	− 0.097	0.137	0.477	0.907
pCO_2_ at discharge	0.152	0.077	**0.049**	1.164
BMI *Z*-score at discharge	− 1.256	0.759	0.098	0.285
Constant	− 176.020	155.048	0.256	0.000

*BMI* body mass index, *pCO*_*2*_, partial pressure of carbon dioxide, *b* regression coefficient, *S.E.* standard error of regression coefficient, *OR* odds ratio

pCO_2_ at both admission and discharge and age were significantly correlated (at admission, *r* = 0.18 *p* = 0.03; at discharge, *r* = 0.24 *p* = 0.01). pCO_2_, upon admission and at discharge, and time from initial behaviors related to AN were not significantly correlated (upon admission, *r* = 0.042 *p* = 0.64; at discharge, *r* = 0.04 *p* = 0.67)

Bold indicates* p* < *0.05*

Findings from scheduled measurements of electrolytes, including serum potassium and phosphorous, remained within the normal range for all participants. No refeeding syndrome cases were identified.

No significant differences were found between males and females. Similarly, there were no significant differences found between the AN-R and AN-BP subtypes.

## Discussion

The results of this study indicate that hypercapnia may be used as a risk factor for readmission in adolescents with AN complications leading to hospitalization. This association is observed both when hypercapnia is considered as a dichotomous variable (normocarbia/hypercapnia) and when taken as a continuous variable. Moreover, the pattern did not depend on the AN subtype or participant sex. The correlation between age and pCO_2_ is clinically unimportant. To our knowledge, no other laboratory alterations have been associated to AN-related readmission.

Readmissions related to AN are an indicator of suboptimal disease course. In cohorts of children and adolescents, a readmission rate for AN complications of between 20 and 45% has been reported [43–46]. AN and, specifically, AN-related hospitalization lead to a worse quality of life for the hospitalized person and their siblings and caregivers [47, 48].

Male sex, low socioeconomic status, concurrent diseases and nutritional status have been reported to play a role in the treatment outcome and odds of readmission [38, 46, 49]. Specifically, lower BMI at discharge from inpatient care is related to increased odds of relapse and poor clinical outcome in adult patients [50–53]. Total body fat proportion and a central adiposity distribution pattern have also been associated with long-term outcome and risk of relapse [54–56]. Despite this existing evidence, no laboratory markers have been associated with AN outcome. Early recognition of the signs of clinical worsening during outpatient follow-up is important to detect and treat early complications and reduce the risk of readmission. Electrolyte imbalance and acid–base disturbances are more frequent in persons diagnosed with AN. These findings may be used as red, prompting clinicians to conduct a differential diagnosis with an ED. In a population-based case–control study, persons diagnosed with an ED were significantly associated with a twofold greater likelihood of electrolyte abnormalities in the previous month [57]. Hypokalemia, hyperkalemia, hyponatremia, hypernatremia, hypophosphatemia and metabolic alkalosis were associated with a higher risk of an ED in this study [57]. This outcome is consistent with the classical laboratory abnormalities described in previous publications [21, 58, 59]. However, these inferences are based on adults diagnosed with AN, who tend toward higher rates of laxative or diuretics misuse and are more likely to present purging behaviors. Preteens and adolescents with AN are less likely to misuse drugs and engage in purging behaviors [60], so biochemical alterations in these age groups may differ from those of their older counterparts. We found no abnormal electrolyte concentrations, including serum potassium and phosphorous, despite the marked malnutrition and rapid weight loss that a significant segment of participants showed upon admission.

Some studies about younger participants have described normal blood gas findings and abnormal respiratory patterns [61, 62]. Kerem et al*.* first reported respiratory acidosis in a limited sample of adolescents hospitalized for recent onset of AN [22]. In these patients, acidosis improved during hospitalization and was related to BMI and HR evolution [22]. Pulmonary function testing revealed no major alterations whatsoever [63]. Our results reinforce the hypothesis according to which hypercapnia and respiratory acidosis may be a common finding in children and adolescents with a diagnosis of AN and admitted for medical stabilization regardless of the onset of the disorder. Furthermore, our study shows that the magnitude of hypercapnia is associated with the risk of AN-related readmission.

Remarkably, pH, pCO_2_ and bicarbonate concentration were stable throughout the follow-up period, this despite the notable weight increase and prolonged hospitalization. One possible explanation is that our study was not limited to recently diagnosed participants, as half of the participants had been showing signs of AN for over a year before admission. The persistence of acid–base disorders is controversial, as some studies report that the anomalies disappear [22, 63] while another state that they persist despite weight gain [64]. We hypothesize that respiratory muscle weakening, especially diaphragm performance, may play a role in evolving AN over the longer term. A slower and more superficial respiratory pattern and a diminished response to hypercapnia due to an increased vagal tone has been reported in persons with recent-onset AN [61, 65]. More research is needed to evaluate the extent of these abnormalities and their association with the clinical outcome.

### Strengths and limits

The prospective design, remarkably large number of participants and prolonged follow-up period are clear strengths of the present study.

However, our results should be interpreted considering the limitations of the study. The most significant limitation is that the study was carried out in one specialist ED unit, which could hinder the generalizability of the study findings. Besides, no control group of patients was included in the study, so no comparison between groups is possible.

Another limitation concerns the use of venous samples for blood gas analysis, as reference ranges and equivalence to arterial values have not been set [66, 67]. Furthermore, venous blood samples are preferable to arterial blood for standard pediatric care [68], as the latter are more painful and require an additional puncture, so an analysis of arterial blood gases was not considered appropriate. In addition, pulmonary function tests were not performed, as no abnormal findings were reported in previous studies [63].

Finally, an additional limitation is the inclusion of participants at different stages of AN and ages, as in many pediatric cohorts. Although age was not a clinically significant variable in our study, pathophysiologic mechanisms may vary greatly from a prepuberal child to a person nearing adulthood.

## Conclusion

Hypercapnia and respiratory acidosis are common findings in children and adolescents hospitalized due to AN. This alteration generally persists for at least 6 months after discharge, despite clinical amelioration. Altered venous blood gas values should be thoroughly considered when reassessing the physiologic impact of AN, as pCO_2_ elevation at discharge is associated with higher odds of readmission. To our knowledge, no findings from laboratory tests have been reported to date as potential predictors of readmission. Further research is needed to fully understand the mechanism of hypercapnia and its consequences for patients with AN, as well as to determine objective predictors of an unfavorable disease course in outpatients.

### What is already known on this subject?

Persons diagnosed with AN often develop electrolyte disturbances and acid–base imbalances. Specifically, previous studies have shown that respiratory acidosis and hypercapnia are common in children and adolescents hospitalized due to AN decompensation. However, patients presenting these abnormalities have not been previously linked to a poorer outcome than those with values within normal ranges.

### What this study adds?

This study shows that hypercapnia at hospital discharge is associated with higher odds of readmission. Interestingly no previous research has linked any laboratory test results to the risk of hospital readmission.

These findings will hopefully provide evidence for further studies to describe the mechanism and consequences of hypercapnia and respiratory acidosis in patients with a diagnosis of AN.

## Data Availability

The datasets analyzed during the current study are available from the corresponding author on reasonable request.

## Abbreviations

AN: Anorexia nervosa
AN-BP: Binge-eating and purging anorexia nervosa
ANOVA: Analysis of variance
AN-R: Restricting anorexia nervosa
BMI: Body mass index
DBP: Diastolic blood pressure
DSM-5: Diagnostic and Statistical Manual of Mental Disorders, fifth edition
ED: Eating disorder
EC G: Electrocardiogram
HC${\text{O}}_{3}^{ - }$: Bicarbonate concentration
HR: Heart rate
IQR: Interquartile range
M: Mean
pCO_2_: Partial pressure of carbon dioxide
SBP: Systolic blood pressure
SD: Standard deviation
*t*_0_: Assessment upon admission
*t*_1_: Assessment at discharge
*t*_2_: Assessment six months after discharge
